# The Sociological Context of Incarceration and Health

**DOI:** 10.1017/jme.2023.65

**Published:** 2023

**Authors:** Jason Schnittker

**Affiliations:** 1: UNIVERSITY OF PENNSYLVANIA, PHILADELPHIA, PA, USA.

**Keywords:** Incarceration, Punishment, Reintegration, Prison Healthcare, Chronic Disease

In “Risk Reduction Policies to Reduce HIV in Prisons,” Das, Ladha, and Klitzman^1^ focus on four programs to reduce intra-prison transmission of HIV and enhance treatment. All four programs are worthwhile, even with the obstacles enacting such initiatives would entail.

It is valuable, though, to consider the larger sociological context of the relationship between incarceration and health and some additional obstacles to reform. In particular, I see two types of risk, especially if one is interested in improving the general health of those involved with the criminal justice system. A focus on HIV, though absolutely critical, might overshadow other health issues that also ought to be important in any discussion of enhancing care. In addition, a focus on improving the treatment or prevention of illness in prison can obscure the process by which incarceration is linked to health. The connection between incarceration and health has multiple dimensions, elevating the significance of criminal justice contact — and all the processes that contact entails—to a fundamental cause of disease. Such a relationship necessitates a different type of focus, over multiple environments.

Enhancing medical care in prisons is desirable and needed, but if the goal is improving the health of those with criminal justice contact, a focus on treatment in prison is limited. Up to half of people in prison have a chronic illness, a rate far higher than in the general population, but relatively few have HIV.^2^ Three times more have tuberculosis than HIV, and far more have hypertension. Furthermore, there are differences among custodial institutions. The authors define “prisons” broadly, as is appropriate, but there are significant differences between jails, state prisons, and federal prisons, both in opportunities and obstacles. Incarceration in jail is short-term, incarceration in prisons is much longer, though the median time is still short of two years.^3^ To an approximation, the quality of health care overlaps with the average length of a sentence: jails provide grossly inadequate care, state prisons provide somewhat better care, and federal prisons a bit better still. If the goal is enhancing care among underserved communities, the high turnover and volume of jails provides the best opportunity, though reform in state and especially federal prisons is more feasible, as their administration is focused more on long-term custody and, by extension, treatment. Much is made of the stock size of the prison population, but over 600,000 people are released from jail and prison every year.^4^ In addition, many states have initiated processes of rather rapid decarceration in recent years. The US still incarcerates far too many people, but the needs of recently released people are pressing and not terribly well accounted for when considering the inadequacies of care in prison.

In addition, some calls for reform overlook the headwinds flowing not from politics but from the law. As the authors note, people in prison are the only people in the US guaranteed access to care. Yet setting the mandate to care in the context of avoiding cruel and unusual punishment puts strong constraints on the focus of that care and, furthermore, sets it apart from the standards of care employed outside of prison. The treatment of HIV fits well within the prohibition against cruel and unusual punishment in the sense that, absent treatment, HIV-infected people risk “lingering death,” as the Supreme Court has interpreted the Eighth Amendment. Similarly, people with active transmittable infections risk infecting others, potentially elevating the cruelty of their punishment. Prison care is, in many ways, focused precisely on what the courts have compelled prisons to do. For instance, prisons have (at least recently) done well in detecting and treating tuberculosis, as failing to do so can result in major outbreaks and within-prison spread.^5^ What the Eighth Amendment does not compel prisons to do is to detect and treat every condition relevant to the functioning of the people in its care. It does not require them to adequately treat major depression. It does not require prisons to vigorously detect and treat new cases of hypertension, as the risk is likely to emerge only years after release. And it does not require them to detect or treat many other sexually transmitted infections, apart from those that directly risk serious complications or death. Prisons treat what they are obligated treat, during the window in which they are obligated to do so. They avoid cruel and unusual punishment while people are in their custody but they do not embrace a broad ethic of care.

It is valuable, though, to consider the larger sociological context of the relationship between incarceration and health and some additional obstacles to reform. In particular, I see two types of risk, especially if one is interested in improving the general health of those involved with the criminal justice system. A focus on HIV, though absolutely critical, might overshadow other health issues that also ought to be important in any discussion of enhancing care. In addition, a focus on improving the treatment or prevention of illness in prison can obscure the process by which incarceration is linked to health. The connection between incarceration and health has multiple dimensions, elevating the significance of criminal justice contact — and all the processes that contact entails—to a fundamental cause of disease. Such a relationship necessitates a different type of focus, over multiple environments.

Advancing a more robust standard of care is structurally difficult, for all the reasons the authors articulate, but it will also require two philosophical pivots. For one, it will require advancing a different conception of health. When care is defined vis-à-vis the avoidance of punishment, it necessarily assumes negative rather than positive connotations. Good health is, however, a capacity as much as a state, and a more positive conception is coherent with a concept of justice. We expect people released from prison to restore their place in society, and good health is necessary for second chances. The challenges of finding a job with a criminal record, for instance, are many, and depression can thwart the best intentions and the strongest motivation. We expect parents released from prison to provide for their children, but doing so is difficult when their own health requires routine attention. Chronic pain can be treated while incarcerated, but freedom from pain is necessary for full civic participation after release.

A narrow focus on treatment in prisons can also overshadow the social determinants of health, as well as the gaps created by the institutions involved in criminal justice. Much of the evidence suggests that the highest risk for mortality is after release. This risk of mortality is especially high in the first two-weeks, but the elevated risk of illness persists long after release.^6^ Much of the elevated risk does not reflect poor access to care or gaps in coverage, but rather from the enduring consequences of a criminal record.^7^

The consequences of incarceration are not limited to infectious disease. The cruelty of incarceration stems as much from the conditions of reentry as those of confinement. Elevating the health of people released from prisons involves ensuring better social reintegration, including securing housing, finding employment, and encouraging broader participation in civic society. People in prison are strictly separated from society — a condition fundamental to what the criminal justice system regards as punishment — but there is much to be gained from allowing them the standard of care afforded others. A shared standard of care can help to bring them back.
